# Antioxidant, Anti-Inflammatory, and Cytotoxic Properties and Chemical Compositions of *Filipendula palmata* (Pall.) Maxim.

**DOI:** 10.1155/2021/6659620

**Published:** 2021-02-15

**Authors:** Hongyin Zhang, Guangzhe Li, Rongxin Han, Rongrong Zhang, Xintong Ma, Miao Wang, Shuai Shao, Mingming Yan, Daqing Zhao

**Affiliations:** ^1^Changchun University of Chinese Medicine, Changchun 130117, China; ^2^Jilin Province Technology Innovation Center of Traditional Chinese Medicine Health Food, Changchun University of Chinese Medicine, Changchun 130117, China

## Abstract

*Filipendula palmata* (Pall.) Maxim. remains unexplored and underutilized resources with a high potential to improve human health. In this study, a new ursane-type triterpenoid, namely, 2*α*, 3*β*-dihydroxyurs-12-en-28-aldehyde (compound 10), and other 23 known compounds were isolated. 5 triterpenoids (compounds 6, 8, and 10–12), 11 flavonoids (compounds 13–15 and 17–24), 6 phenolic compounds (compounds 1, 2, 4, 5, 9, and 16), 2 sterols (compounds 3 and 7) were isolated from the aqueous solution extract of the aerial parts of *F. palmata*. The structures of all compounds were elucidated by the use of extensive spectroscopic methods such as infrared spectroscopy (IR), high-resolution electrospray ionization mass spectrometry (HR-ESI-MS), ^1^H-NMR, and ^13^C-NMR. The solvent extractions of ethyl acetate fraction were evaluated for antioxidant activities using DPPH (2, 2-diphenyl-1-picrylhydrazyl) and ABTS^+^ (2, 2′-azino-bis (3-ethylbenzothiazoline-6-sulfonic acid)) methods. The anti-inflammatory effects of the compounds were evaluated in lipopolysaccharide- (LPS-) stimulated RAW 264.7 macrophages. The extract cytotoxicity on the cancer cell lines MCF-7, HeLa, 4T1, and A549 was determined by MTT assay. As a result, compounds 10, 11, and 12 exhibited better antioxidant activity compared to the other compounds. Compounds 8–24 had different inhibitory effects on the release of NO, TNF-*α*, and IL-6 in LPS-stimulated RAW 264.7 cells. The new compound has shown a significant inhibiting effect on cancer cells, and the cell inhibition rate increased in a dose-dependent manner. Further research to elucidate the chemical compositions and pharmacological effects of *F. palmata* is of major importance towards the development and foundation of clinical application of the species.

## 1. Introduction

With the improvement of people's health awareness, different kinds of healthcare products are gradually developed. Herbal tea, one of the popular beverages consumed worldwide, has the widest applicability, second only to drinking water. The human uses of herbal teas are believed to have originated around thousands years ago in China, and its efficacy and taste are closely related to the specific components of plants. Nowadays, various kinds of herbal tea have become the most common beverage and food in people's daily life. Meadowsweet tea is one of the most popular herbal tea products, and its raw material comes from the genus *Filipendula*. The use of some *Filipendula* genus as herbal medicines is known, and the aerial parts and roots have a significant pharmaceutical interest, being used by daily consumption in Russia and other Siberia countries as wild tea. *Filipendula* is one of the most popular perennial herbaceous plant genera for herbal tea preparation, and it includes many significant plants of the family Rosaceae and more than 20 varieties are distributed all over the world (http://www.ncbi.nlm.nih.gov/taxonomy/). These herbs are used due to the specific honey-like fragrance of the flowers and the pleasant taste of water decoctions. Among them, the most studied species are *Filipendula ulmaria* (L.) Maxim. (meadowsweet) and *Filipendula vulgaris* Moench (dropwort), which are officinal plant species in many countries. In these species, researchers have found different classes of bioactive constituents including salicylates, phenolic acids, flavonoids and flavonoid glycosides, and tannins [1–9]. Recently, Katanic [10] showed that *F. ulmaria* extracts were effective in reducing kidney oxidative stress and mitigating tissue damage. The same extracts attenuated the genotoxicity of cisplatin in a reverse dose-dependent manner and did not demonstrate any in vitro cytotoxic activity at all the applied concentrations. Bespalov [11] showed that meadowsweet (*F. ulmaria*) decoction was able to inhibit colorectal carcinogenesis induced by the methylnitrosourea in rats. The chemical composition of meadowsweet had a statistically significant decrease in the overall tumor incidence and multiplicity by 1.4 and 2.9 times. Samardzic et al. [12] reported that meadowsweet and dropwort were rich in polyphenols that belong to the classes of flavonol glycosides, phenolic acids, and hydrolysable tannins as a folk medicine for their antirheumatic, antipyretic, and antiulcer properties.

*F. palmata* belongs to *Filipendula* genus, which is widely distributed in Northeast China, usually called by Siberian meadowsweet. *F. palmata* was the northeast genuine drug in China, species commonly used to treat gout, rheumatism, epilepsy, frostbite, burn, and gynecologic hemostasis. Recently, *F. palmata* had been considerably less studied plant, its chemical compositions had been reported only with essential oils; moreover, the biological activity of *F. palmata* had not yet been reported. The objectives of this study were to verify the pharmacological activity of drugs and to find a new substitute with better efficacy in *Filipendula* genus.

## 2. Materials and Methods

### 2.1. Plant Materials

*F. palmata* (dried and finely ground) were obtained from raw materials collected in Northeast China and identified by Professor Dacheng Jiang, Changchun University of Chinese Medicine. A voucher specimen has been deposited in Changchun University of Traditional Chinese Medicine in Changchun, China.

### 2.2. Chemicals and Reagents

All reagents and standards were of analytical, LC-MS, and HPLC grade. Formic acid (98%), methanol (99.8%), and LC-MS grade acetonitrile were purchased from Sigma-Aldrich (Steinheim, Germany). IR spectra were recorded using a Bruker Vertex 70 Fourier Transform Infrared (FT-IR) Spectrometer (Bruker, Germany) with KBr disks. ^1^H-NMR, ^13^C-NMR, distortionless enhancement by polarization transfer (DEPT), ^1^H-^1^H correlated spectroscopy (^1^H-^1^H COSY), heteronuclear multiple quantum correlation (HMQC), and heteronuclear multiple bond correlation (HMBC) experiments were performed on a Bruker AVANCE 600 spectrometer (Bruker, Germany; 600 MHz for ^1^H-NMR and 150 MHz for ^13^C-NMR), TMS was used as an international standard and DMSO-d6 as a solvent. High-performance liquid chromatography (HPLC) was performed using an Agilent 1260 Series HPLC system (Agilent, USA) equipped with four pumps with an in-line degasser, autosampler, oven, and ultraviolet detector (UVD). HR-ESI-MS was measured on IonSpec 7.0 T Fourier Transform Ion Cyclotron Resonance Mass Spectrometry (FT-ICR-MS) spectrometer (Bruker, USA). Column chromatography was performed with silica gel (200–300 mesh) (Qingdao Marine Chemical Factory, China). Thin-layer chromatography (TLC) was carried out with glass precoated silica gel plates (Qingdao Marine Chemical Factory, China). Sephadex LH-20 was used for the column chromatography (Pharmacia, 25–100 *µ*m). Ultrapure water was produced using a water purifier (Si chuan, China). All spectrophotometric measurements were performed on UV-VIS double beam spectrophotometer UV-2700 (Shimadzu, Japan). 2, 2-Diphenyl-1-picrylhydrazyl (DPPH, Sigma, USA) and 2, 2′-azino-bis (3-ethylbenzothiazoline-6-sulfonic acid) (ABTS^+^, Sigma, USA) were used for analysis. The absorbance was measured using a microplate reader (PowerWave XS2, BioTek Instruments Inc., USA).

### 2.3. Cell Lines

Human cervical cancer cells HeLa, human breast adenocarcinoma cells MCF-7, mouse breast cancer cells 4T1, and human lung cancer cells A549 were purchased from American Type Culture Collection (Manassas, VA) and cultured in DMEM (Gibco, USA) supplemented with 10% fetal bovine serum (Gibco, USA), 2 mmol/L L-glutamine, 100 U/ml penicillin, and 100 *μ*g/ml streptomycin at 37°C and in an atmosphere of 5% CO_2_ and 95% humidity. The culture medium containing 0.1% DMSO was used as the samples (specimens and controls).

The mice macrophage RAW264.7 (American Type Culture Collection, Manassas, VA, USA) cell line was maintained in DMEM supplemented with 10% heat-inactivated fetal bovine serum (FBS), penicillin G (100 units/mL), streptomycin (100 mg/mL), and L-glutamine (2 mM). The cells were grown in a humidified atmosphere containing 5% CO_2_ at 37°C.

### 2.4. Extraction and Isolation

**Figure 1:**
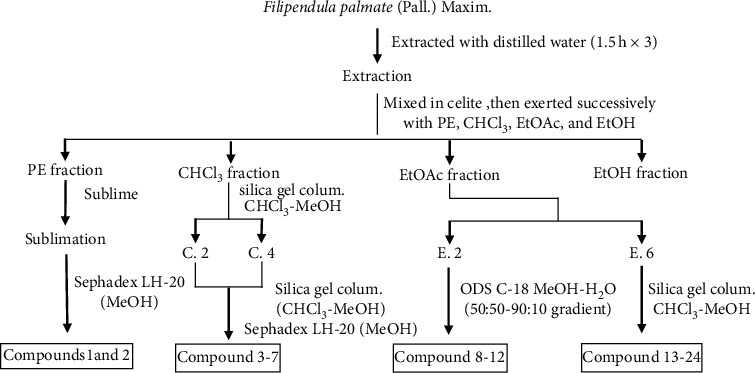
The extraction process route of compounds 1–24.

The air-dried aerial parts of *F. palmata* (50 kg) were powdered and macerated with distilled water (1 : 14 w/v) for 12 hours at room temperature and then extracted three times (1.5 h each) with distilled water. After filtration, filtrates were merged, aqueous extracts were evaporated on a rotary evaporator (vacuum rotary evaporator), obtained aqueous extracts (1105 g) were then mixed with diatomite, and successively partitioned with petroleum ether, chloroform, ethyl acetate, and ethanol to obtain the petroleum ether fraction (289 g), chloroform fraction (183 g), ethyl acetate fraction (121 g), and ethanol fraction (309 g). The petroleum ether was removed, and the extraction part of petroleum ether becomes a thick paste, which used the sublimation method and was subjected to LH-20 eluting with MeOH to give compounds 1 and 2. The chloroform soluble fraction was chromatographed on silica gel column eluting with CHCl_3_/MeOH in gradient (40 : 1 to 1 : 1) and then detected by TLC and the same components were merged to obtain 5 fractions (C. 1–C. 5); C. 2 and C.4 fractions were chromatographed on silica gel column eluting with CHCl_3_/MeOH and detected by TLC, and the same components were merged, which were separated by Sephadex LH-20 column to give compounds 3–7. The ethyl acetate soluble fraction was dissolved in methanol. After filtration, fractions were separated by a LH-20 gel and eluted with methanol and the same components were combined by TLC detection, to give 6 fractions (E.1–E.6); E.2 fraction was separated by octadecylsilyl (ODS) reverse-column chromatography and eluted with a gradient of methanol-water (50 : 50–90 : 10) to give compounds 8–12 and E.6 fraction was repeated by silica gel column chromatography and gel LH-20 purification to give compounds 13–24. The ethanol fraction was reserved for reserve (Figure 1).

#### 2.4.1. Paeonol

Colorless acicular crystal, mp: 49–50°C. ^1^H-NMR: *δ*: 7.548 (1H, d, *J* = 9 Hz), *δ*6.364 (1H, dd, *J* = 9, 2.5 Hz), *δ*6.334 (1H, d, *J* = 2.5 Hz), *δ*3.760 (3H, s), *δ*2.477 (3H, s).^13^C-NMR: *δ*: 201.55 (C-1), 159.31 (C-2), 156.32 (C-3), 121.35 (C-4), 105.21 (C-5), 108.13 (C-6), 102.04 (C-7), 56.64 (C-8), 28.29 (C-9). FeCl_3_ reaction showed positive results. It has the same Rf value and retention time compared with a paeonol reference substance for the same TLC and HPLC tests.

#### 2.4.2. Salicylic Acid

White acicular crystal, mp: 158–160°C. ^1^H-NMR: *δ*: 6.86 (1H, dd, H-5), 6.87 (1H, d, H-3), 7.26 (1H, td, H-4), 7.30 (1H, dd, H-6); ^13^C-NMR: *δ*: 112.89 (C-1), 161.09 (C-2), 117.04 (C-3), 135.6 (C-4), 119.13 (C-5), 130.22 (C-6), 171.86 (C-7).

#### 2.4.3. *β*-Daucosterol

White solid powder, mp: 292–294°C. The Liebermann–Burchard test showed positive, purple red color by 10% purple vitriol spray test. It has the same Rf value in a TLC test compared with a reference substance.

#### 2.4.4. Ferulic Acid

White crystal, mp: 170–178°C. ^1^H-NMR: *δ*: H6.92 (2H, s, H-2, 6), 9.31 (3H, brs, OH); ^13^C-NMR: *δ*: 121.2(C-1), 109.2 (C-2), 145.9(C-3), 138.4(C-4), 145.9(C-5), 109.2(C-6), 168.1(C-7). UV test showed blue fluorescence at 365 nm, and the melting point did not decrease after mixing with the ferulic acid standard.

#### 2.4.5. Gallic Acid

White acicular crystal, mp: 236–240°C. ^1^H-NMR: *δ*: H6.92 (2H, s, H-2, 6), 9.31(3H, brs, OH); ^13^C-NMR: *δ*: 121.2(C-1), 109.2(C-2), 145.9(C-3), 138.4(C-4), 145.9(C-5), 109.2(C-6), 168.1(C-7). The Rf value of the compound was the same as that of the standard gallic acid, and the melting point remained unchanged after mixing with gallic acid.

#### 2.4.6. *α*-Amyrin

Colorless acicular crystal, mp: 180–182°C. The Liebermann–Burchard reaction was positive and the Molish reaction was negative. The color of 10% sulfuric acid-ethanol was purple. The Rf value and spot color were consistent with the standard product of *α*-amyrin.

#### 2.4.7. *β*-Sitosterol

White sheet crystal, mp: 138–140°C. The Liebermann–Burchard reaction was positive. The Rf value was consistent with the standard product of *β*-sitosterol in three solvent systems.

#### 2.4.8. Oleanolic Acid

White powder, mp: 306–308°C. ^1^H-NMR: *δ*: 5.30 (1H, d, *J* = 8.3 Hz, H-12), 3.12 (1H, d, *J* = 9.8 Hz, H-3), 1.14 (3H, s, H-27), 0.99(3H, s, H-25), 0.90(3H, s, H-29), 0.88(3H, s, H-23), 0.85(3H, s, H-24), 0.77(3H, s, H-26); ^13^C-NMR: *δ*: 38.5 (C-1), 27.3 (C-2), 77.2 (C-3), 38.3 (C-4), 55.2 (C-5), 18.4 (C-6), 32.8 (C-7), 39.4(C-8), 47.5(C-9), 37.0(C-10), 23.0(C-11), 122.0(C-12), 143.6(C-13), 41.8(C-14), 27.6(C-15), 23.3(C-16), 46.1 (C17), 41.3(C-18), 45.9(C-19), 30.9 (C-20), 33.2 (C-21), 32.2 (C-22), 28.5 (C-23), 15.0 (C-24), 15.8(C-25), 16.7(C-26), 26.1(C-27), 180.1(C-28), 33.2(C-29), 22.8 (C-30). The Rf value was consistent with the standard product of oleanolic acid in several TLC systems, and the melting point does not decrease after mixing with a standard product.

#### 2.4.9. Caffeic Acid

White powder, ^1^H-NMR: *δ*: 9.53 (1H, s), 9.14 (1H, s), 7.42 (1H, d, *J* = 16.0 Hz), 7.03(1H, d, 2.0 Hz), 6.97 (1H, dd, 2 Hz, 8 Hz), 6.73 (1H, d, 8.0 Hz), 6.17 (1H, d, 16.0 Hz); ^13^C-NMR: *δ*: 126.2 (C-1), 115.1 (C-2), 146.0 (C-3), 148.6 (C-4), 115.6 (C-5), 121.6 (C-6), 145.1 (C-7), 116.2 (C-8), 168.3 (C-9). The Rf value was consistent with the standard product of caffeic acid, and the melting point does not decrease after mixing with a standard product.

#### 2.4.10. 2*α*, 3*β*-Dihydroxyurs-12-en-28-aldehyde

Colorless needle crystal, mp: 204–206°C. The Liebermann–Burchard reaction was positive. The molecular formula was C_30_H_48_O_3_ from HR-ESIMS (measured value: *m*/*z* 495.32543 M^+^, calculated value: 495.324052). IR (KBr) *ν*max: 3406, 2812, 2720, 1693, 1640 cm^−1^. For ^1^H-NMR and ^13^C-NMR spectral data, see Table 1.

#### 2.4.11. 2*α*, 3*β*, 19*α*, 23-Tetrahydroxylurs-12-en-28-oic Acid

White powder, ^1^H-NMR: *δ*: 5.41 (1H, H-12), 3.72 (1H, m), 3.61 (1H, d, *J* = 11.2 Hz, H-23a), 3.66 (1H, m), 3.44 (1H, d, *J* = 11.2 Hz, H-23b), 2.61 (1H, s, H-18), 1.53(3H, s), 1.20(3H, s), 1.14(3H, s), 0.99(3H, d, *J* = 6.6 Hz, H-30), 0.91(3H, s), 0.83(3H, s). ^13^C-NMR: *δ*: 49.01(C-1), 70.22(C-2), 78.54(C-3), 44.72(C-4), 48.55(C-5), 19.75(C-6), 33.76(C-7), 41.57(C-8), 49.64(C-9), 40.71(C-10), 25.32(C-11), 130.49(C-12), 140.64(C-13), 43.73(C-14), 30.66(C-15), 27.69(C-16), 50.63(C17), 55.46(C-18), 74.37(C-19), 43.67(C-20), 27.88(C-21), 40.01(C-22),66.78(C-23), 14.56C-24), 17.77(C-25), 17.86(C-26), 25.34(C-27), 181.06(C-28), 27.86(C-29), 16.97(C-30).

#### 2.4.12. Pedunculoside

Colorless acicular crystal, mp: 205–206°C. ^13^C-NMR: *δ*: 37.55 (C-1), 27.86(C-2), 74.93(C-3), 42.98(C-4), 48.67(C-5), 18.78(C-6), 33.66(C-7), 41.56(C-8), 48.33(C-9), 39.86(C-10), 25.33(C-11), 129.62(C-12), 140.55(C-13), 43.62(C-14), 30.78(C-15), 26.69(C-16), 49.06(C-17), 54.97(C-18), 72.53(C-19), 42.86(C-20), 26.79(C-21), 37.88(C-22), 68.87(C-23), 12.68(C-24), 16.72(C-25), 17.69(C-26), 25.15(C-27), 177.81(C-28), 27.95(C-29), 16.88(C-30), 96.33(C-1′), 74.64(C-2′), 79.05(C-3′), 71.78(C-4′), 80.31(C-5′), 62.67(C-6′).

#### 2.4.13. Quercetin

Yellow powder crystal, mp: 315–317°C. ^1^H-NMR: *δ*: 6.17 (1H, d, *J* = 2.0 Hz, H-6), 6.37 (1H, d, *J* = 2.0 Hz, H-8), 6.87 (1H, d, *J* = 8.0 Hz, H-50), 7.62 (1H, dd, *J* = 2.0, 7.5 Hz, H-6), 7.73 (1H, d, *J* = 2.0 Hz, H-20). The magnesium hydrochloride reaction showed rose-red color (positive), and the FeCl_3_ reaction showed dark green. The Rf value was consistent with the standard product of quercetin, and the melting point does not decrease after mixing with a standard product.

#### 2.4.14. Quercitrin

Yellow powder, mp: 181–183°C. ^1^H-NMR: *δ*: 12.652 (1H, s, OH-5), 10.853 (1H, s, OH-7), 9.32 (1H, s, OH-3′), 9.688 (1H, s, OH-4′), 7.295 (1H, d, H-2′), 6.857 (1H, d, H-5′), 7.26 (1H, dd, H-6′), 5.251 (1H, brs, H-1″), 6.206 (1H, d, H-6), 6.386 (1H, d, H-8). ^13^C-NMR: *δ*: 148.39 (C-2), 134.18 (C-3), 177.70 (C-4), 161.25 (C-5), 98.63 (C-6), 164.13 (C-7), 93.56 (C-8), 156.40 (C-9), 104.04 (C-10), 121.05 (C-1′), 115.40 (C-2′), 145.16 (C-3′), 148.39 (C-4′), 115.60 (C-5′), 120.68 (C-6′).

#### 2.4.15. Kaempferol

Yellow powder, mp: 274–280°C. ^1^H-NMR: *δ*: H 6.19(1H, s, H-6), 6.43(1H, s, H-8), 8.04(2H, d, *J* = 8.4 Hz, H-2′, 6′), 6.93(2H, d, *J* = 8.4 Hz, H-3′, 5′), 12.48(1H, brs, OH), 10.12(2H, brs, OH). ^13^C-NMR: *δ*: 147.22(C-2), 136.14(C-3), 176.44(C-4), 161.22(C-5), 98.87(C-6), 164.71(C-7), 94.06(C-8), 156.73(C-9), 103.47(C-10), 122.25(C-1′), 129.94(C-2′, 6′), 115.93(C-3′, 5′), 159.74(C-4′). The magnesium hydrochloride reaction was positive, and the Rf value was consistent with the standard product of kaempferol.

#### 2.4.16. Ellagic Acid

Yellow powder, mp: 360–368°C. ^1^H-NMR: *δ*: 7.81 (2H, s, H-5, 5′). ^13^C-NMR: *δ*: 160.22(C-7, 7′), 149.41(C-4, 4′), 140.65(C-3, 3′), 136.77(C-2, 2′), 112.98(C-1, 1′), 110.83(C-5, 5′), 107.15(C-6, 6′).

#### 2.4.17. Kaempferol-3-O-*α*-L-rhamnoside

Yellow powder, mp: 256.5–258.5°C. ^1^H-NMR: *δ*: 12.75 (1H, s, 5-OH), 11.08(1H, s, 7-OH), 10.51(1H, s, 4′-OH), 6.61(1H, d, *J* = 2.0 Hz, 6-H), 6.91 (1H, d, *J* = 2.0 Hz, H-8), 7.82(2H, d, *J* = 8.8 Hz, 2′, 6′-H), 7.32(2H, d, *J* = 8.8 Hz, 3′, 5′-H), 5.65(1H, s, H-1″), 0.91(3H, d, *J* = 5.6 Hz, -CH3). ^13^C-NMR: *δ*: 17.22(C-6″), 70.32(C-5″), 70.41(C-2″), 70.77(C-3″), 71.33(C-4″), 93.88(C-8), 98.98(C-6), 102.03(C-1″), 104.65(C-10), 115.78(C-3′,C-5′), 120.67(C-1′), 130.78(C-2′, C-6′), 134.56(C-3), 156.94(C-2), 157.44(C-9), 160.24(C-4′), 161.65(C-5), 164.67(C-7), 177.88(C-4).

#### 2.4.18. Kaempferitrin

Yellow acicular crystal, mp: 207–209°C. ^1^H-NMR: *δ*: 1277(1H, s, 5-OH), 10.54(1H, s, 7-OH), 6.75 (1H, d, *J* = 2.0 Hz, 6-H), 6.91(1H, d, *J* = 2.0 Hz, 8-H), 7.88(2H, d, *J* = 8.8 Hz, 2′, 6′-H), 7.05(2H, d, *J* = 8.8 Hz, 3′, 5′-H), 5.76(1H, s, H-1″), 5.46(1H, s, H-1″), 1.27(3H, d, *J* = 5.2 Hz, 7-rha-CH3), 1.02(3H, d, *J* = 5.2 Hz, 3-rha-CH3). ^13^C-NMR: *δ*: 17.75(C-6″), 17.98(C-6‴), 69.78(C-5″), 70.51(C-5‴), 70.61(C-3″), 70.63(C-3‴), 70.65(C-2″), 70.77(C-2‴), 71.32(C-4″), 71.56(C-4‴), 94.56(C-8), 98.34(C-6), 99.44(C-1″), 101.39(C-1‴), 105.88(C-10), 115.44(C-3′, C-5′), 120.73(C-1′), 130.67(C-2′, C-6′), 134.85(C-3), 156.81(C-2), 157.38(C-9), 160.78(C-4′), 160.97(C-5), 161.65(C-7), 177.89(C-4).

#### 2.4.19. Kaempferol-3-O-rutinoside

Yellow powder, mp: 171.5–173.5°C. ^1^H-NMR: *δ*: 13.16(1H, s, 5-OH), 11.78(1H, s, 7-OH), 11.21(1H, s, 4′-OH), 6.51(1H, d, *J* = 2.0 Hz, 6-H), 6.91(1H, d, *J* = 2.0 Hz, 8-H), 8.23(2H, d, *J* = 8.8 Hz, 2′, 6′-H), 6.94(2H, d, *J* = 8.8 Hz, 3′, 5′-H), 5.67(1H, d, *J* = 7.6 Hz, 1″-H), 4.56(1H, d, *J* = 1.2 Hz, 1‴-H), 1.12(3H, d, *J* = 6.0 Hz, -CH3). ^13^C-NMR: *δ*: 18.87(C-6‴), 67.58(C-6″), 68.64(C-5‴), 70.14(C-4″), 71.03(C-2‴), 71.06(C-3‴), 72.08(C-4‴), 74.03(C-2″), 75.21(C-5″), 76.36(C-3″), 93.58(C-8), 99.07(C-6), 100.84(C-1‴), 101.97(C-1″), 104.55(C-10), 115.67(C-3′, C-5′), 121.25(C-1′), 131.24(C-2′, C-6′), 133.07(C-3), 156.75(C-2), 156.98(C-9), 159.55(C-4′), 161.37(C-5), 164.87(C-7), 177.54(C-4).

#### 2.4.20. Isoquercitrin

Yellow powder, mp: 233–235°C. ^1^H-NMR: *δ*: 12.85(1H, s, 5-OH), 10.97(1H, s, 7-OH), 9.88(1H, brs, 3′-OH), 9.52(1H, brs, 4′-OH), 6.41(1H, d, *J* = 2.0 Hz, 6-H), 6.81(1H, d, *J* = 2.0 Hz, 8-H), 7.81(2H, dd, *J* = 2.0 Hz, 8.4 Hz, 2′, 6′-H), 6.99(1H, d, *J* = 8.4 Hz, 5′-H), 5.72(1H, d, *J* = 7.6 Hz, 1″-H). ^13^C-NMR: *δ*: 61.01(C-6″), 70.09(C-4″), 74.24(C-2″), 76.54(C-3″), 77.87(C-5″), 93.67(C-8), 98.24(C-6), 101.24(C-1″), 104.88(C-10), 115.72(C-2′), 116.82(C-5′), 121.74(C-1′), 121.56(C-6′), 133.72(C-3), 144.55(C-3′), 148.74(C-4′), 156.42(C-2), 156.23(C-9), 161.42(C-5), 164.51(C-7), 177.54(C-4).

#### 2.4.21. Apigenin

Yellow powder, mp: 345–347°C. ^1^H-NMR: *δ*: 13.52 (1H, s, 5-OH), 8.63(2H, d, *J* = 8.8 Hz, H-2′,6′), 7.53(2H, d, *J* = 8.8 Hz, H-3′, 5′), 7.42(1H, s, H-3), 6.92(1H, d, *J* = 2.0 Hz, H-8), 6.57(1H, d, *J* = 2.0 Hz, H-6). ^13^C-NMR: *δ*: 182.17(C-4), 164.61(C-7), 163.87(C-5), 161.74(C-2), 161.51(C-4′), 157.62(C-9), 128.77(C-2′,C-6′), 121.56(C-1′), 116.12(C-3′,C-5′), 104.24(C-3), 103.84(C-10), 99.22(C-6), 94.35(C-8).

#### 2.4.22. Rutoside

Yellow powder, mp: 210–217°C. ^1^H-NMR: *δ*: 12.77 (1H, s, OH-5), 10.92(1H, s, OH-7), 9.76(1H, s, OH-4′), 9.88(1H, s, OH-3′), 7.89(1H, d, *J* = 8.0 Hz, H-6′), 7.67(1H, s, H-2′), 6.88 (d, 1H, *J* = 8.0 Hz, H-5′), 6.78(1H, d, *J* = 2.4 Hz, H-8), 6.69(1H, d, *J* = 2.4 Hz, H-6), 5.57(1H, d, *J* = 7.6 Hz, H-1″), 4.58(1H, d, *J* = 1.2 Hz, H-1‴), 1.22(3H, d, *J* = 6.0 Hz, CH3). ^13^C-NMR: *δ*: 177.36(C-4), 164.52(C-7), 161.76(C-5), 156.46(C-9), 156.53(C-2), 148.78(C-4′), 144.58(C-3′), 133.34(C-3), 121.98(C-6′), 121.21(C-1′), 116.27(C-5′), 115.52(C-2′), 103.19(C-10), 101.32(C-1″), 100.77(C-1‴), 98.56(C-6), 93.35(C-8), 76.44(C-3″), 75.79(C-5″), 75.38(C-2″), 74.80(C-4‴), 71.48(C-4″), 70.36(C-2‴), 70.33(C-3‴), 69.79(C-5‴), 68.52(C-6″), 17.77(C-6‴).

#### 2.4.23. Avicularin

Yellow powder, mp 214–217°C. ^1^H-NMR: *δ*: 5.82 (1H, s, H-1″), 6.51(1H, d, *J* = 1.2 Hz, H-6), 6.61(1H, d, *J* = 1.2 Hz, H-8), 7.51(1H, d, *J* = 6.8 Hz, H-5′), 7.98(1H, dd, *J* = 1.2,6.8 Hz, H-6′), 8.66(1H, d, *J* = 1.2 Hz, H-2′), 9.78(1H, s, OH-3′), 10.13(1H, s, OH-4′), 11.12(1H, s, OH-7), 12.86(1H, s, OH-5). ^13^C-NMR: *δ*: 158.04(C-2), 134.27(C-3), 180.34(C-4), 162.99(C-5), 99.50(C-6), 165.71(C-7), 94.34(C-8), 158.67(C-9), 105.11(C-10), 121.84(C-1′), 115.61(C-2′), 145.67(C-3′), 149.21(C-4′), 116.39(C-5′), 122.77(C-6′), 108.75(C-1″), 82.66(C-2″), 78.01(C-3″), 87.67(C-4″), 61.98(C-5″).

#### 2.4.24. Hyperoside

Yellow crystal, mp 235–236°C. ^1^H-NMR: *δ*: 5.49 (1H, d, *J* = 7.6 Hz, H-1″), 6.44(1H, brs, H-6), 6.68(1H, brs, H-8), 6.96(1H, d, *J* = 8.0 Hz, H-5′), 7.71(1H, d, *J* = 1.2 Hz, H-2′), 7.90(1H, dd, *J* = 1.2, 8.4 Hz, H-6′), 9.64(1H, s, OH-3′), 9.91(1H, s, OH-4′), 11.08(1H, s, OH-7), 12.93(1H, s, OH-5). ^13^C-NMR: *δ*: 157.22(C-2), 134.94(C-3), 178.31(C-4), 161.92(C-5), 99.87(C-6), 165.18(C-7), 94.92(C-8), 157.43(C-9), 106.78(C-10), 122.08(C-1′), 115.84(C-2′), 145.24(C-3′), 149.15(C-4′), 116.61(C-5′), 122.43(C-6′), 102.65(C-1″), 71.82(C-2″), 73.85(C-3″), 68.77(C-4″), 76.25(C-5″), 60.57(C-6″).

### 2.5. Bioactivity

#### 2.5.1. Antioxidant Activity

*(1) DPPH assay*. The methods for determining DPPH free radical scavenging activity were analyzed by Hu's method with slight modifications [13]. About 0.2 mL of tested compounds at various concentrations were added to 2 mL DPPH solution (21.4 *µ*g/mL ethanol solution), respectively. The mixture, protected from light, was reacted for 30 min. The decrease in absorbance was monitored at 517 nm, to obtain absorbance *A*_*i*_. The control was the DPPH solution. The sample solution was replaced with 70% ethanol solution, to obtain *A*_0_, and the DPPH was used to obtain *A*_*j*_ in the same method.

  % radical scavenging activity = 1−[(*A*_*i*_–*A*_*j*_)/*A*_0_] × 100%
 
*A*_0_: absorption of 2 mL DPPH 70% ethanol solution and 2 mL 70% ethanol solution.
 
*A*_*i*_: absorption of 2 mL DPPH 70% ethanol solution and 2 mL sample.
 
*A*_*j*_: absorption of 2 mL 70% ethanol solution and 2 mL sample.

*(2) ABTS* ^*+*^ *assay.* The methods for determining ABTS^+^ free radical scavenging activity were analyzed by Biao's method with slight modifications [14]. About 0.2 mL of tested compounds at various concentrations was added to 2 mL ABTS^+^ solution, respectively. The mixture, protected from light, was reacted for 30 min. The decrease in absorbance was monitored at 734 nm. The control was 0.2 mL of distilled water and 2 mL of ABTS^+^ solution.

The same method was used in vitamin C (Vc). The half-maximal inhibitory concentration (IC_50_) was used to evaluate the ABTS^+^ free radical scavenging activity and the DPPH free radical scavenging activity.

#### 2.5.2. Anti-Inflammatory Activity

RAW264.7 cells were seeded in 96-well plates at a density of 8 × 10^4^ cells/well for 24 h. The cells were randomly divided into control group, LPS (1 *µ*g/mL) group, and LPS (1 *µ*g/mL) + compound 8–24 (50 *µ*g/mL) group. After adding the corresponding drug, the supernatant was used to detect NO, TNF-*α*, and IL-6 after culturing for 24 h at 5% CO_2_ and 37°C under saturated humidity [15].

#### 2.5.3. Cytotoxicity Assay

Cell proliferation was measured using the colorimetric MTT method for HeLa, 4T1, A549, and MCF-7 cells [16]. Compounds 8–24 were dissolved in DMEM culture media containing 0.1% DMSO at final concentrations of 0–400 *μ*g/mL. The HeLa, 4T1, A549, and MCF-7 cells were grown in 96-well plates at 9 × 10^3^ cells per well, incubated at 37°C for 24 h, and then treated with various concentrations of compound 10 for 48 h. The control cells were exposed to culture media containing 0.1% DMSO. Then, 50 mL of MTT solution (5 mg/mL) was added to each well. Cells were incubated for three additional hours. Finally, 150 mL of DMSO was added to dissolve the formed crystals. The absorbance at 570 nm was measured by scanning with a microplate reader. The experiment was repeated 3 times. Calculation of the impact of drugs on cell growth inhibition rate and IC_50_ values is performed with the following equation:

(1) $$\begin{array}{c} \text{growth inhibition rate}\left(100\%\right)=\frac{\left(D_{0}-D_{1}\right)}{D_{0}}\times 100\%, \end{array}$$

where *D*_0_ is the OD value of the control wells and *D*_1_ is the OD value of the sample wells.

### 2.6. Statistical Analysis

Statistical analyses were performed using SPSS 21.0 for the variance of the experimental data. The results were expressed by X ± s. *P* < 0.05 was considered to have statistical significance.

## 3. Results and Discussion

### 3.1. Identification of Chemical Compositions

**Figure 2:**
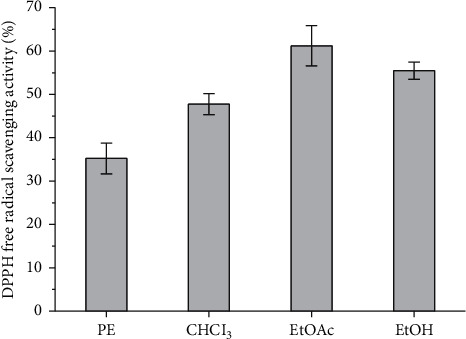
Preliminary study on DPPH activity of petroleum ether, chloroform, ethyl acetate, and ethanol fractions.

The contents of the major groups of phenolic compounds (flavonoids, tannins, catechins, and proanthocyanidins) were determined for the phytochemical characterization of *F. palmata* (Pall.) Maxim., as shown in Table 2. It is obvious from the presented results that *F. palmata* was richer in the total phenolic compounds (277.21 mg GAE g^−1^) and flavonoid and flavonol contents (32.98 and 22.12 mg RUE g^−1^, respectively). In this study, preliminary activity studies were conducted on the petroleum ether, chloroform, ethyl acetate, ethanol, and other parts separated from the water extract of *F. palmata*. It was found that the ethyl acetate and ethanol fractions have good antioxidant activity (Figure 2). In order to more reasonably test the effective active part of *F. palmata*, this experiment selected the ethyl acetate part for the compound activity investigation.

**Figure 3:**
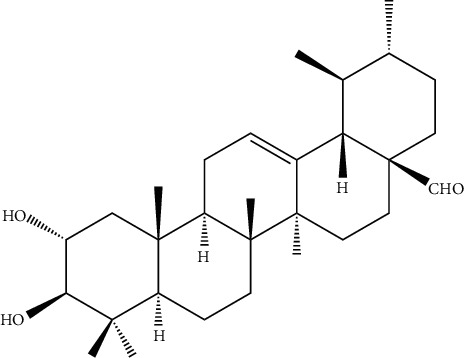
Structures of the new compound 10.

The aqueous extract of colorless needle crystal was isolated by column chromatographic (CC) fractionation to give compound 10 (Figure 3), together with 24 known compounds: paeonol (compound 1) [17], salicylic acid (compound 2) [18], *β*-daucosterol (compound 3) [19], ferulic acid (compound 4) [20], gallic acid (compound 5) [19], *α*-amyrin (compound 6) [21], *β*-sitosterol (compound 7) [22], oleanolic acid (compound 8) [23], caffeic acid (compound 9) [24], 2*α*, 3*β*, 19*α*, 23-tetrahydroxylurs-12-en-28-oic acid (compound 11) [25], pedunculoside (compound 12) [26], quercetin (compound 13) [19], quercitrin (compound 14) [27], kaempferol (compound 15) [28], ellagic acid (compound 16) [29], kaempferol-3-O-*α*-L-rhamnoside (compound 17) [30], kaempferitrin (compound 18) [31], kaempferol-3-O-rutinoside (compound 19) [32], isoquercitrin (compound 20) [33], apigenin (compound 21) [34], rutoside (compound 22) [35], avicularin (compound 23) [36], and hyperoside (compound 24) [27].

Characteristics of compound 10 are as follows: It is a white amorphous powder, its IR spectrum exhibited absorption bands due to -OH at 3406 cm^−1^, –CHO at 2812 cm^−1^, 2720 cm^−1^, and 1693 cm^−1^, and double bond at 1640 cm^−1^. The HR-ESI-MS of compound 10 indicated the molecular formula C_30_H_48_O_3_ (*m*/*z* 495.32543 M^+^, 495.324052). The ^1^H-NMR spectra (Table 1) showed seven methyl hydrogen signals at *δ*: 0.70 (3H, S), 0.76(3H, S), 0.81(3H, d), 0.86(3H, d), 0.91(3H, S), 0.92(3H, S), and 1.03(3H, S), one hydrogen signal of tri-substituted olefin bond at 5.09 (1H, brs), and one aldehyde group signal at *δ*: 8.45. The ^13^C-NMR and the DEPT spectra (Table 1) showed seven methyl carbon signals at *δ*: 16.27, 16.40, 17.08, 17.13, 17.13, 21.17, and 23.18, two tertiary carbon signals *δ*: 123.86 and 138.73, two methylene carbon signals of continuous oxygen at *δ*: 67.09, 82.22, and one aldehyde signal at *δ*: 166.49. Combining the IR, ^1^H-NMR, ^13^C-NMR, and DEPT spectra's data suggested that an ursane-type triterpenoid moiety existed in the structure of compound 10. Compared with the reference which published a compound methyl, 2*α*, 3*β*-dihydroxyurs-12-en-28-oate, it was showed that all data except C-28 were consistent. Based on the above information, it was confirmed that C-28 was an aldehyde group. In summary, the structure of compounds is inferred to be 2*α*, 3*β*-dihydroxyurs-12-en-28-aldehyde.

### 3.2. Biological Activity Results

#### 3.2.1. Antioxidant Activity

The ability of plants to show antioxidant activity is owing to their composition and a mixture of different antioxidants, mainly polyphenolic compounds with different action mechanisms. Because of their synergistic interactions, it is indispensable to use several methods in order to determine the in vitro antioxidant capacity of plant extracts [37]. DPPH and ABTS^+^ scavenging capacity are the most commonly used methods for determining antioxidation performance in vitro. The DPPH and ABTS^+^ radical scavenging activities were performed to evaluate the antioxidant capacities of the different active fractions compared with ascorbic acid (vitamin C), which was served as a control. The semi-inhibitory concentration (IC_50_) of compounds 8–24 for ABTS^+^ and DPPH· free radical scavenging activity is shown in Table 3. Compared with other active components, compound 10 has better antioxidant activity, which is related to the structure of ursane-type compounds [38]. Another possibility is that the compound containing aldehyde group was more likely to undergo oxidation reactions and have better antioxidant capacity [39]. Previous studies have shown that the antioxidant activities of different *Filipendula* extractions have been confirmed. In the process of activity comparison of related subgenus plants, the crude extracts from different sources have excellent antioxidant activities [40].

#### 3.2.2. Anti-Inflammatory Activity

Macrophages are necessary to maintain the body's immune balance and play an important role in the host's resistance to pathogen infection [41]. In the process of regulating immunity, stimulating activated macrophages can release some immune regulatory factors. Proinflammatory cytokines have been used as biomarkers for the development and progression of inflammation in macrophage models, such as NO, IL-6, and TNF-*α*. Lipopolysaccharide (LPS) is a component of the cell wall of Gram-negative bacteria. It is an endotoxin. When it acts on macrophages, it stimulates the Toll-like receptors on the cell membrane of the host cell to secrete inflammatory factors. The mechanism is to change the expression of NO, TNF-*α*, and IL-6 via the NF-*κ*B pathway, which was induced by lipopolysaccharides [42, 43]. Therefore, we evaluated the effects of different compounds on mouse macrophages (RAW 264.7) induced by LPS. In the inflammatory response of RAW264.7 cells stimulated by LPS, compounds 8–24 had different inhibitory effects on the release of NO, TNF-*α*, and IL-6 in LPS-stimulated RAW 264.7 cells and showed good anti-inflammatory activity in vitro, in which compound 10 was effective against inflammatory factors. The results are shown in Table 4. Among plants of the same genus, meadowsweet and dropwort have been confirmed to have good antihyperlipidemia and antiedema activity in the rat inflammation model induced by carrageenan injection, and both displayed good safety profiles [44]. In a word, *F. palmata* exerts its anti-inflammatory activity in RAW264.7 cells stimulated by lipopolysaccharide by inhibiting the production of nitric oxide and proinflammatory cytokines.

#### 3.2.3. Cytotoxic Activity

**Figure 4:**
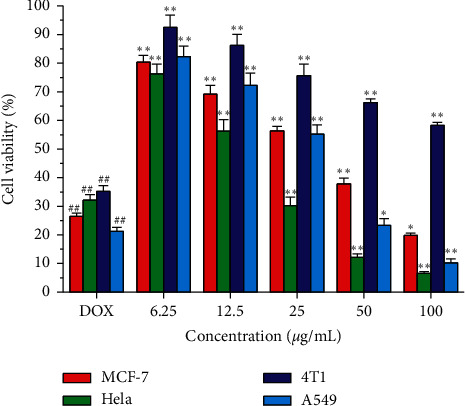
The cell inhibition rate of compound 10 of HeLa, MCF-7, 4T1, and A549 cells. (^##^*p* < 0.01 vs. control group; ^*∗∗*^*p* < 0.01 vs. model group).

Compounds 8–24 isolated from ethyl acetate fraction were examined for their antiproliferative activity towards four cancer cell lines: MCF-7, HeLa, 4T1, and A549 cell lines. The MTT colorimetric assay was adopted to assess the antiproliferative activity as described by Mosmann [16]. Doxorubicin was used as a control in this assay. As shown in Table 5, the results were expressed as median growth inhibitory concentration (IC_50_) values that represent the compound concentration required to produce a 50% inhibition of cell growth after 48 h of incubation. The results of the MTT assay showed that compounds 10, 11, and 12 can significantly inhibit the growth of tumor cells MCF-7, 4T1, A549, and HeLa with IC_50_ value. A549 and HeLa cells were found to be more sensitive to the impact of the tested compounds than MCF-7 and 4T1 cells, except compound 10 which is more effective towards HeLa cells. On the other hand, as shown in Figure 4, after 48 h of compound 10 treatments, the proliferation of HeLa, 4T1, A549, and MCF-7 cells was obviously inhibited and the inhibition rate was increased dose-dependently within the concentration ranges tested, and when the concentration of compound 10 reached 100 *μ*g/mL, it led to a significant increase in MCF-7, 4T1, A549 and HeLa cell growth inhibitions, which were 19.85%, 6.55%, 58.21%, and 10.22%.

A large number of studies had reported that triterpenoids have potential antitumor activity, including examples of lupane-, oleanane-, and ursane-type triterpenoids. Previous studies had confirmed that ursane-type triterpenoids have extensive anti-inflammatory, antibacterial, antifungal, and antitumor activities [45–48]. However, the antitumor effect of ursane-type triterpenoids from the *F. palmata* had not been reported, especially the anticervical cancer effect of ursane-type triterpenoid compound 10. In this experiment, the effect of ursane-type triterpenoid compound 10 on human cervical cancer HeLa cells and human breast adenocarcinoma MCF-7 cells had been further confirmed, suggesting that the compound has a certain antihuman cervical cancer potential. On the other hand, it is speculated that *F. palmata* is often used to repel mosquitoes in summer in Northeast China, which may be related to the chemical constituents contained in *F. palmata* and needs further study and confirmation.

## 4. Conclusion

In this study, we use *F. palmata* as our research object. We found a new ursane-type triterpenoid compound, after extraction and separation by column chromatography, namely, 2*α*, 3*β*-dihydroxyurs-12-en-28-aldehyde (compound 10), and other 23 compounds, based on the spectroscopic analysis. Most of the compounds from *F. palmata* possessed antioxidant, anti-inflammatory, and antitumor effects. In addition to the new compounds found, compounds 11, 12, and 24 were first isolated from this genus and confirmed that each had significant biological activity by detection. The results suggested that *F. palmata* extraction was a potent natural antioxidant and antineoplastic, it was a new natural functional plant source with high content of healthy ingredients, and it would become a new beverage source of plant raw materials or a more efficient substitute for herbal tea. Further study on isolation and identification of more bioactive compounds from *F. palmata* will be helpful to understand this important herbal medicine.

## Figures and Tables

**Table 1:** The ^1^H-NMR (600 MHz, in DMSO-d6) and ^13^C-NMR (150 MHz, DMSO-d6) data for compound 10.

No.	HMBC		HMQC	
	^13^C-NMR	^1^H-NMR	2*J*	3*J*
1	46.81			
2	67.09	3.42(1H, brs)	C_3_	
3	82.21	2.74(1H, brs)	C_2_	C_23,_ C_24_
4	40.03			
5	54.73	0.73(1H, brs)		C_1_
6	18.01			
7	32.73			
8	39.80			
9	47.04			
10	38.84			
11	22.91			
12	123.86	5.09(1H, brs)		
13	138.73			
14	41.71			
15	27.61			
16	24.00			
17	47.07			
18	52.54	2.15(1H, brs)		
19	40.98			
20	38.61			
21	30.48			
22	36.52			
23	28.80	0.91(3H, S)		C_3,_ C_5_
24	17.13	0.70(3H, S)		C_3,_ C_5,_ C_23_
25	16.27	0.92(3H, S)		C_5_
26	17.08	0.76(3H, S)		C_9,_ C_14_
27	23.19	1.03(3H, S)		C_15_
28	166.49	8.45(1H, brs)		
29	18.0	0.81(3H, d, *J* = 8.4, H-29)		
30	21.18	0.86(3H, d, *J* = 87.8, H-30)		

**Table 2:** General phytochemical characteristics of *F. palmata* (Pall.) Maxim.

Plant	Total phenolic content (mg GAE g^−1^)	Total phenolic acids (mg CAE g^−1^)	Flavonoid content (mg RUE g^−1^)	Flavonol content (mg RUE g^−1^)	Condensed tannin content (mg GAE g^−1^)	Gallotannin content (mg GAE g^−1^)
*F. palmata* (Pall.) Maxim.	277.21 ± 8.25	60.32 ± 2.11	32.98 ± 2.13	22.12 ± 1.86	202.21 ± 10.45	43.31 ± 3.05

Data are represented as means ± SD (*n* = 3). GAE: gallic acid equivalents; CAE: caffeic acid equivalents; RUE: rutin equivalents.

**Table 3:** The IC_50_ of the different active fractions for 2, 2′-azino-bis (3-ethylbenzothiazoline-6-sulfonic acid) (ABTS ^+^) and 2, 2-diphenyl-1-picrylhydrazyl (DPPH) free radical scavenging activity.

	The IC_50_ of ABTS^+^ free radical scavenging activity (*μ*g/mL)	The IC_50_ of DPPH free radical scavenging activity (*μ*g/mL)
Vitamin C	60.65 ± 1.25	45.58 ± 0.25
8	90.42 ± 2.12^*∗∗*^	102.23 ± 1.12^*∗∗*^
9	77.99 ± 1.31^*∗∗*^	65.05 ± 0.87^*∗∗*^
10	62.16 ± 1.45^*∗∗*^	58.07 ± 1.01^*∗∗*^
11	70.05 ± 1.21^*∗∗*^	93.56 ± 2.35^*∗∗*^
12	75.25 ± 1.55^*∗∗*^	89.27 ± 1.88^*∗∗*^
13	132.34 ± 3.44^*∗∗*^	172.23 ± 3.56^*∗∗*^
14	85.57 ± 2.52^*∗∗*^	160.11 ± 4.02^*∗∗*^
15	91.23 ± 2.65^*∗∗*^	150.25 ± 3.22^*∗∗*^
16	102.87 ± 3.11^*∗∗*^	147.56 ± 4.12^*∗∗*^
17	132.12 ± 4.54^*∗∗*^	160.23 ± 2.35^*∗∗*^
18	141.24 ± 2.88^*∗∗*^	180.51 ± 2.65^*∗∗*^
19	121.13 ± 4.01^*∗∗*^	170.24 ± 4.21^*∗∗*^
20	88.56 ± 2.97^*∗∗*^	155.64 ± 3.46^*∗∗*^
21	106.21 ± 2.05^*∗∗*^	165.33 ± 2.22^*∗∗*^
22	150.14 ± 3.47^*∗∗*^	182.01 ± 3.37^*∗∗*^
23	116.74 ± 2.99^*∗∗*^	159.09 ± 5.12^*∗∗*^
24	157.36 ± 3.84^*∗∗*^	188.22 ± 4.25^*∗∗*^

^##^ *p* < 0.01 vs. control group; ^*∗∗*^*p* < 0.01 vs. model group. The values are means ± standard errors of the experiment carried out in triplicate.

**Table 4:** Effects of compounds on NO, TNF-*α*, and IL-6 in LPS-stimulated RAW264.7 cells.

	NO (*µ*mol/mL)	TNF-*α* (ng/mL)	IL-6 (ng/mL)
Control	4.67 ± 0.07	10.25 ± 0.85	0.12 ± 0.02
LPS	8.12 ± 0.18^##^	49.13 ± 2.01^##^	0.72 ± 0.03^##^
LPS + 8	7.36 ± 0.41^*∗*^	41.25 ± 1.55^*∗∗*^	0.63 ± 0.03^*∗∗*^
LPS + 9	7.12 ± 0.14^*∗*^	35.66 ± 1.25^*∗∗*^	0.51 ± 0.01^*∗∗*^
LPS + 10	5.52 ± 0.26^*∗∗*^	28.82 ± 2.31^*∗∗*^	0.32 ± 0.03^*∗∗*^
LPS + 11	6.01 ± 0.45^*∗∗*^	38.36 ± 1.63^*∗∗*^	0.39 ± 0.03^*∗∗*^
LPS + 12	6.98 ± 0.14^*∗∗*^	35.12 ± 1.15^*∗∗*^	0.35 ± 0.09^*∗∗*^
LPS + 13	6.28 ± 0.26^*∗∗*^	32.41 ± 2.40^*∗∗*^	0.48 ± 0.01^*∗∗*^
LPS + 14	7.12 ± 0.34^*∗∗*^	35.53 ± 1.25^*∗∗*^	0.42 ± 0.02^*∗∗*^
LPS + 15	6.01 ± 0.12^*∗∗*^	32.45 ± 1.85^*∗∗*^	0.53 ± 0.01^*∗∗*^
LPS + 16	7.63 ± 0.21^*∗∗*^	40.32 ± 1.43^*∗∗*^	0.42 ± 0.01^*∗∗*^
LPS + 17	5.86 ± 0.15^*∗∗*^	30.26 ± 1.08^*∗∗*^	0.44 ± 0.02^*∗∗*^
LPS + 18	6.10 ± 0.25^*∗∗*^	31.58 ± 1.64^*∗∗*^	0.46 ± 0.02^*∗∗*^
LPS + 19	7.21 ± 0.14^*∗∗*^	33.77 ± 1.71^*∗∗*^	0.54 ± 0.02^*∗∗*^
LPS + 20	5.38 ± 0.15^*∗∗*^	40.35 ± 2.06^*∗∗*^	0.51 ± 0.03^*∗∗*^
LPS + 21	6.37 ± 0.31^*∗∗*^	44.41 ± 2.51^*∗∗*^	0.43 ± 0.02^*∗∗*^
LPS + 22	7.69 ± 0.13^*∗∗*^	35.15 ± 1.26^*∗∗*^	0.62 ± 0.03^*∗∗*^
LPS + 23	7.28 ± 0.35^*∗∗*^	46.41 ± 2.03^*∗∗*^	0.46 ± 0.02^*∗∗*^
LPS + 24	5.88 ± 0.12^*∗∗*^	34.68 ± 1.46^*∗∗*^	0.50 ± 0.08^*∗∗*^

^##^ *p* < 0.01 vs. control group; ^*∗∗*^*p* < 0.01 vs. model group. The values are means ± standard errors of the experiment carried out in triplicate.

**Table 5:** Antiproliferative activities of compounds against four tumor cell lines (IC_50_, *µ*g/mL).

Compound	The IC_50_ of antiproliferative activity (*μ*g/mL)			
	MCF-7	HeLa	4T1	A549
Doxorubicin	65.61 ± 2.25	12.52 ± 1.02	152.22 ± 7.25^*∗∗*^	10.72 ± 1.11^*∗∗*^
8	96.58 ± 3.69^*∗∗*^	46.21 ± 3.33^*∗∗*^	205.15 ± 8.77^*∗∗*^	39.88 ± 2.05^*∗∗*^
9	125.66 ± 4.77^*∗∗*^	50.84 ± 4.21^*∗∗*^	181.27 ± 6.99^*∗∗*^	45.55 ± 1.37^*∗∗*^
10	68.13 ± 3.54^*∗∗*^	13.22 ± 1.33^*∗∗*^	172.22 ± 8.12^*∗∗*^	29.09 ± 1.22^*∗∗*^
11	72.32 ± 2.25^*∗∗*^	16.55 ± 1.25^*∗∗*^	195.66 ± 10.21^*∗∗*^	26.52 ± 1.54^*∗∗*^
12	75.65 ± 3.21^*∗∗*^	18.17 ± 1.63^*∗∗*^	230.11 ± 12.11^*∗∗*^	33.55 ± 1.62^*∗∗*^
13	80.25 ± 4.12^*∗∗*^	19.22 ± 2.30^*∗∗*^	245.22 ± 10.69^*∗∗*^	52.21 ± 1.39^*∗∗*^
14	81.36 ± 2.66^*∗∗*^	22.36 ± 1.89^*∗∗*^	285.66 ± 9.87^*∗∗*^	46.36 ± 1.77^*∗∗*^
15	83.64 ± 3.10^*∗∗*^	22.57 ± 2.06^*∗∗*^	>400	55.30 ± 2.12^*∗∗*^
16	110.95 ± 6.21^*∗∗*^	38.24 ± 2.55^*∗∗*^	235.66 ± 13.25^*∗∗*^	39.55 ± 1.25^*∗∗*^
17	85.66 ± 4.12^*∗∗*^	21.21 ± 1.78^*∗∗*^	227.55 ± 11.21^*∗∗*^	40.75 ± 2.36^*∗∗*^
18	88.63 ± 2.12^*∗∗*^	23.77 ± 2.13^*∗∗*^	265.22 ± 16.32^*∗∗*^	29.36 ± 0.25^*∗∗*^
19	86.49 ± 3.82^*∗∗*^	23.84 ± 2.11^*∗∗*^	301.11 ± 18.12^*∗∗*^	35.32 ± 1.89^*∗∗*^
20	82.34 ± 2.65^*∗∗*^	20.96 ± 1.21^*∗∗*^	>400	49.87 ± 1.92^*∗∗*^
21	84.47 ± 3.33^*∗∗*^	23.65 ± 1.84^*∗∗*^	322.02 ± 16.14^*∗∗*^	38.65 ± 2.13^*∗∗*^
22	85.11 ± 3.58^*∗∗*^	22.31 ± 1.64^*∗∗*^	275.22 ± 11.01^*∗∗*^	42.09 ± 1.69^*∗∗*^
23	89.65 ± 2.36^*∗∗*^	24.33 ± 2.12^*∗∗*^	226.22 ± 13.21^*∗∗*^	51.25 ± 2.17^*∗∗*^
24	84.29 ± 3.64^*∗∗*^	21.95 ± 1.76^*∗∗*^	352.21 ± 15.62^*∗∗*^	50.12 ± 1.99^*∗∗*^

^##^ *p* < 0.01 vs. control group; ^*∗∗*^*p* < 0.01 vs. model group. The values are means ± standard errors of the experiment carried out in triplicates.

## Data Availability

The data used to support the findings of this study are included within the article, and the data used to support the findings of this study are available from the corresponding author upon request.
